# Supplementary material for: Identification of Biomarkers Associated with Liver Metastasis Progression from Colorectal Cancer Using Exosomal RNA Profiling
Source: Cancers (Basel). 2022 Sep 28;14(19):4723. doi: 10.3390/cancers14194723 (PMC9562015; doi:10.3390/cancers14194723)

Figures S1. Progression free survival by in exosome and GSE 41258 by CXCL10, CXCL11 in mCRC with liver metastasis

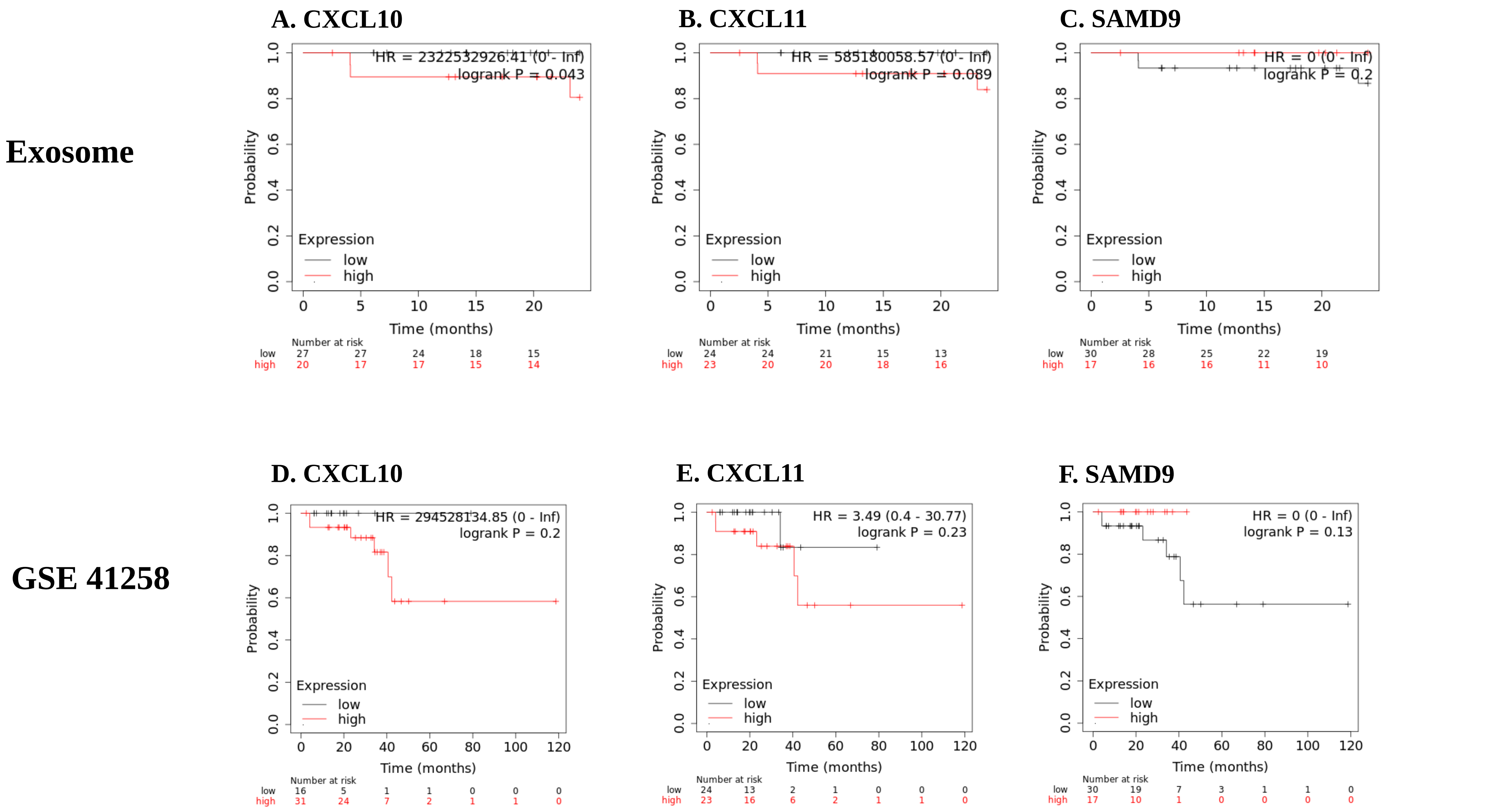

Supplement: Supplementary file 1 [file cancers-14-04723-s001.zip › cancers-1792625-supplementary.pdf]
